# The association between how medical students were selected and their perceived stress levels in Year-1 of medical school

**DOI:** 10.1186/s12909-023-04411-0

**Published:** 2023-06-16

**Authors:** Vera M.A. Broks, Karen M. Stegers-Jager, Suzanne Fikrat-Wevers, Walter. W. Van den Broek, Andrea M. Woltman

**Affiliations:** Institute of Medical Education Research Rotterdam, Erasmus MC University Medical Centre Rotterdam, Rotterdam, the Netherlands

**Keywords:** Selection methods, Stress perception, Academic performance

## Abstract

**Background:**

The prevalence of medical students’ mental distress is high. While schools apply various methods to select a well-performing and diverse student population, little is known about the association between different selection methods and the well-being of these students during medical school. The present retrospective multi-cohort study assessed whether students selected by high grades, assessment, or weighted lottery showed different stress perception levels in Year-1 of medical school.

**Methods:**

Of 1144 Dutch Year-1 medical students, 650 (57%) of the cohorts 2013, 2014, and 2018 who were selected by high grades, assessment, or weighted lottery completed a stress perception questionnaire (PSS-14). A multilevel regression analysis assessed the association between selection method (independent variable) and stress perception levels (dependent variable) while controlling for gender and cohort. In a post-hoc analysis, academic performance (*optimal* vs. *non-optimal*) was included in the multilevel model.

**Results:**

Students selected by assessment (B = 2.25, *p* < .01, effect size (ES) = small) or weighted lottery (B = 3.95, *p* < .01, ES = medium) had higher stress perception levels than students selected by high grades. Extending the regression model with optimal academic performance (B=-4.38, *p* < .001, ES = medium), eliminated the statistically significant difference in stress perception between assessment and high grades and reduced the difference between weighted lottery and high grades from 3.95 to 2.45 (B = 2.45, *p* < .05, ES = small).

**Conclusions:**

Selection methods intended to create a diverse student population – assessment and lottery - are associated with higher stress perception levels in Year-1 of medical school. These findings offer medical schools insights into fulfilling their responsibility to take care of their students’ well-being.

**Supplementary Information:**

The online version contains supplementary material available at 10.1186/s12909-023-04411-0.

## Introduction

A growing concern for medical schools is the high prevalence of mental distress among medical students compared to their age-matched peers [1, 2]. Mental distress of medical students is associated with both student characteristics and medical school characteristics [3–6]. The selection method of medical schools is a school characteristic that directly influences the composition of cohorts regarding student characteristics, but has not been studied in relation to medical student distress during medical school. The responsibility of medical schools is to accommodate the students they select and, as such, to obtain insight into the stress students experience after enrolment into the program. Therefore, the present study explores the association between selection methods and the stress perception levels of medical students attending medical school.

Stress is negatively associated with short-term and long-term well-being [7–9]. Estimates show that one-quarter to one-third of medical students show depression symptoms [10, 11], and roughly 40% of medical students show burn-out symptoms [12]. Due to this high prevalence of mental distress, associations between the characteristics of medical schools and medical students on one side and the well-being of students on the other have been the subject of a growing body of research [3–6]. An example of a medical school characteristic associated with student well-being is assessment. Assessment in itself [4] and, more specifically, assessment policies with higher performance standards have been shown to increase stress perception levels [13]. By contrast, pass/fail grading can impact the well-being of medical students positively compared to grading with three or more intervals [6]. Examples of medical student characteristics associated with well-being are gender and academic performance. Both female students and lower-performing students show higher levels of psychological distress compared to their male or well-performing peers, respectively [1, 4, 13–15].

Medical schools seek the right methods to select diverse and successful student cohorts to serve societal needs [16, 17]. Medical school selection is generally based on grades, assessment, and/or lottery. Selection based on grades, such as pre-university Grade Point Average (pu-GPA), has been positively linked to academic performance [18–20]. However, grades-based selection is considered too narrow to select students whose competencies align with the medical profession and also fails to ensure a representative student population [17, 21]. Assessment-based selection generally entails an extensive procedure, often including a combination of tests measuring academic and/or non-academic skills [17]. When the assessment-based selection is aligned with the medical school curriculum, it can predict academic performance [22]. However, research indicates that assessment-based selection procedures may still disadvantage minority groups, depending on how the assessment is implemented (e.g., too much focus on academic skills instead of non-academic criteria) [17, 23–27]. An advantage of selection by lottery is that it does not harm the diversity of the student population since no specific selection criteria are applied [28]. However, applying lottery - even when weighted for pre-university grades - may go at the expense of academic performance. Students selected by weighted lottery show lower performance from Year-1 of medical school up until clerkships [18, 29–31]. In sum, the specific selection methods of medical schools have consequences for the characteristics of the selected students.

To ensure that medical schools adequately accommodate their selected students, insights regarding student well-being during the medical school program related to the selection method are required. In the Netherlands, different selection methods coexisted in the past decade: direct selection based on high pre-university grades, assessment-based selection, and lottery weighted for pre-university grades. This coexistence has offered the unique opportunity to explore how students selected with different methods compare regarding their stress perception levels during medical school. The present retrospective multi-cohort study addressed the following research question: *Do differences exist in stress perception levels at the end of Year-1 between medical students selected on the basis of high grades, assessment, or weighted lottery?* In addition, student gender and the medical school assessment policy were controlled for as these factors are known to be associated with stress perception [13]. This explorative study is a first step in providing insight into the association between selection methods and student stress in medical school and will aid medical schools in fulfilling their responsibility concerning the well-being of all their students.

## Methods

### Context

In the Netherlands, since 2000, students were selected for medical school on the basis of a weighted lottery system, or a school-specific assessment, or students had direct access to medical school based on a high pre-university Grade Point Average (pu-GPA) [17]. The premise behind the weighted lottery was that the probability of students being selected increased along with their pre-university Grade Point Average (pu-GPA). From 2017 onward, however, selection by weighted lottery was no longer an option, and medical schools had to choose their own selection method(s).

The present study was conducted at Erasmus MC Medical School as part of an ongoing research program on the effects of assessment changes and student stress [13, 32]. To examine the effect of modified assessment policies, data were collected in cohorts 2013, 2014, 2018, and 2019. The cohort years relate to these assessment changes in medical school. However, due to the COVID-19 outbreak, data from cohort 2019 was unusable for the present study. Therefore, in the present study, students from the cohorts of 2013, 2014, and 2018 were included. Thus, although there was some time between data collection, this data collection did allow for a multi-cohort study in which students were also still admitted to medical school via lottery. Every year, a fixed number of places were available for students to start medical school. In the present study, we distinguish students selected on the basis of high grades, assessment, or weighted lottery (see Additional file 1). In all three cohorts, some of the students were selected based on high grades and assessment. For cohorts 2013 and 2014, some of the students were selected by weighted lottery. Given that high grades provided direct access to medical school, students selected by assessment or weighted lottery had lower pre-university grades than those directly admitted. After selecting the students with high pre-university grades, a pre-defined maximum percentage of students was selected by assessment, and the remaining places were filled with students selected by lottery (for cohorts 2013 and 2014). Selection by assessment consisted of CV/extracurricular activities and study skills tests [33], and – for cohort 2014 and 2018 - pu-GPA (if available). Students were ranked based on their performance on these assessment tools, and the best-ranked students were selected.

At the Erasmus MC Medical School, the Academic Dismissal (AD) policy in Year-1 was different for cohorts 2013, 2014, and 2018. Students from cohort 2013 had to obtain 67% of Year-1 credits in Year-1 (67% AD policy), resulting in academic probation if they could not obtain the required credits. After two years, 100% of Year-1 credits had to be attained, or academic dismissal would follow. From cohort 2014 onwards, the AD policy changed in an attempt to better determine at an early stage whether students are suitable for the program [34]. Subsequently, for cohort 2014, performance standards increased to 100% of Year-1 credits in Year-1 (100% AD policy), with compensation possibilities for up to two grades between 5.0 and 5.49 (scale from 1 to 10; 5.5 as a passing grade). This compensation was given on the condition that these grades were not in the same thematic block and that the average grade would not drop below 6.0. From 2017 onwards, the AD policy was adjusted due to the unforeseen side-effect of high numbers of Year-1 repeaters stemming from the 100% AD policy, thereby leaving the medical school with too many Year-1 students. As a result, the performance standards were lowered to 75% of Year-1 credits (75% AD policy). Students from cohort 2018 needed to obtain 75% of Year-1 credits in Year-1 (without compensation possibilities), or academic dismissal would follow. At the same time, no major changes were made in the curriculum between these cohorts.

### Participants and procedure

Year-1 Bachelor students who were selected by grades, assessment, or weighted lottery and who enrolled in medical school in cohort 2013 (385 students), cohort 2014 (382 students), and cohort 2018 (377 students) were invited to complete a questionnaire regarding stress perception levels in May of their first academic year. Data was collected on paper during a lecture. In addition, an online version was available for students who were unable to complete the questionnaire during the lecture. Students provided written informed consent for the questionnaire and agreed to link questionnaire results to relevant data from the student administration. The university student administration provided data regarding students’ cohort (the first year of enrolment), gender (male/female), how they were selected, and their academic performance. The number of students who completed the questionnaire and provided informed consent determined the sample size of the study. We did not perform a minimum sample size calculation. Data regarding student gender and selection method were also analysed on an aggregated level for the complete cohorts to assess the representativeness of the sample. In line with the national regulations on personal data protection, no individual consent was required for the data on the complete cohorts. An exemption was made for individual consent by the privacy office of Erasmus University Rotterdam since the data were analysed on an aggregated level for scientific purposes in the public interest, namely to improve education. This exemption is based on Article 89 of the Algemene Verordening Gegevensbescherming (General Data Protection Regulation; https://www.privacy-regulation.eu/en/89.htm). The study was carried out in accordance with the Declaration of Helsinki and was deemed exempt from review after evaluation by the Medical Ethics Committee of Erasmus MC Rotterdam (MEC-2014-387 and MEC-2019-0448).

### Measurements

#### Stress perception level

Student stress perception levels were measured using the Dutch version of the validated 14-item Perceived Stress Scale (PSS-14) [13, 35] in May of the first academic year, showing a good alpha reliability of 0.871. This questionnaire measures stress perception and a person’s ability to cope with this stress during the last month. Each item, therefore, starts with “In the last month…”. An example is, “In the last month, how often have you felt nervous and stressed?”. All 14 items are scored based on a 5-point Likert scale, which ranges from 0 (never) to 4 (very often). The minimum score on the PSS-14 is 0, and the maximum score is 56.

#### Academic performance (for post-hoc analysis)

For the post-hoc analysis, academic performance was considered because of its previously reported association with selection methods and stress [14, 15]. By including academic performance, we could assess its association with stress perception level and how this association impacted the relationship between selection and stress perception level. Academic performance was measured using a binary variable, indicating whether the students showed optimal academic performance or not. Optimal academic performance was defined as passing all the courses for which the exams (and re-sits, if applicable) had taken place up until the PSS-14 questionnaire was administered.

### Analyses

As a first step, the sample of students who completed the fullPSS-14 questionnaire was compared to the complete cohort with chi-square tests to assess the representability of the sample. A multilevel linear regression analysis with stress perception level as the dependent variable was then performed to control the effects of students (level-1) being nested in different cohorts with different assessment policies (level-2). Cohort was the level-2 variable that needed to be controlled by including random intercepts. Three nested multilevel linear regression models were constructed. Model 1, the null model, included two control variables: the level-2 variable cohort and level-1 variable student gender. In Model 2, the level-1 variable selection method was added to assess the association between selection method and stress perception level. In Model 3, post-hoc analyses were performed by extending Model 2 with the level-1 variable academic performance. This was done to assess its association with stress perception level and its impact on the relation between selection method and stress perception level. The multilevel linear regression models met all assumptions; therefore the analyses were performed without further modifications. The three models were compared by assessing the added value of subsequent models relative to previous ones with AIC, log-likelihood, and ANOVA. For interpretation purposes, effect sizes (ES) were computed for regression coefficients by dividing the regression coefficient by the pooled standard deviation of the PSS-14 for the two groups that were compared. Effect sizes were categorized as small (0.2), medium (0.5), or large (0.8) [36]. All analyses were conducted using RStudio, R version 4.2.1 [37].In line with the STrengthening the Reporting of OBservational studies in Epidemiology (STROBE) statement [38], the STROBE-checklist was completed for this paper (see Additional file 2).

## Results

### Student characteristics

In total, 650 students completed the stress perception questionnaire, resulting in a response rate of 57%. The included cohorts (2013, 2014, 2018) were equally represented in the sample, meaning every cohort represents approximately one-third of the sample (Table 1). 72% of the sample were female students, which percentage was comparable to the complete student cohorts (Table 1). In addition, no statistically significant association was found between cohort and gender (X2 = 3.159, df = 2, p = .206), indicating that the gender distributions were comparable between the different cohorts included. Finally, 51% of the sample was selected by assessment, 40% by high grades, and 9% by weighted lottery. These percentages are representative for the complete student cohorts as confirmed by chi-square tests (Table 1).

**Table 1:** Descriptive statistics of the subgroups included in the study

	Complete cohorts		Sample (RR^*^: 57%)		Sample vs. complete cohorts
	n	%	n	%	
**Cohort**					
**Cohort 2013**	385	(34%)	208	(32%)	X^2^ = 0.684, df = 2, *p* = .711
**Cohort 2014**	382	(33%)	217	(33%)	
**Cohort 2018**	377	(33%)	225	(35%)	
**Gender**					
**Female**	787	(69%)	469	(72%)	X^2^ = 2.072, df = 1, *p* = .150
**Male**	357	(31%)	181	(28%)	
**Selection**					
**High grades**	415	(36%)	261	(40%)	X^2^ = 2.95, df = 2, *p* = .229
**Weighted lottery**	114	(10%)	56	(9%)	
**Assessment**	615	(54%)	333	(51%)	
**Total**	**1144**	**(100%)**	**650**	**(100%)**	

^*^ RR = response rate

### Stress perception

**Fig. 1:**
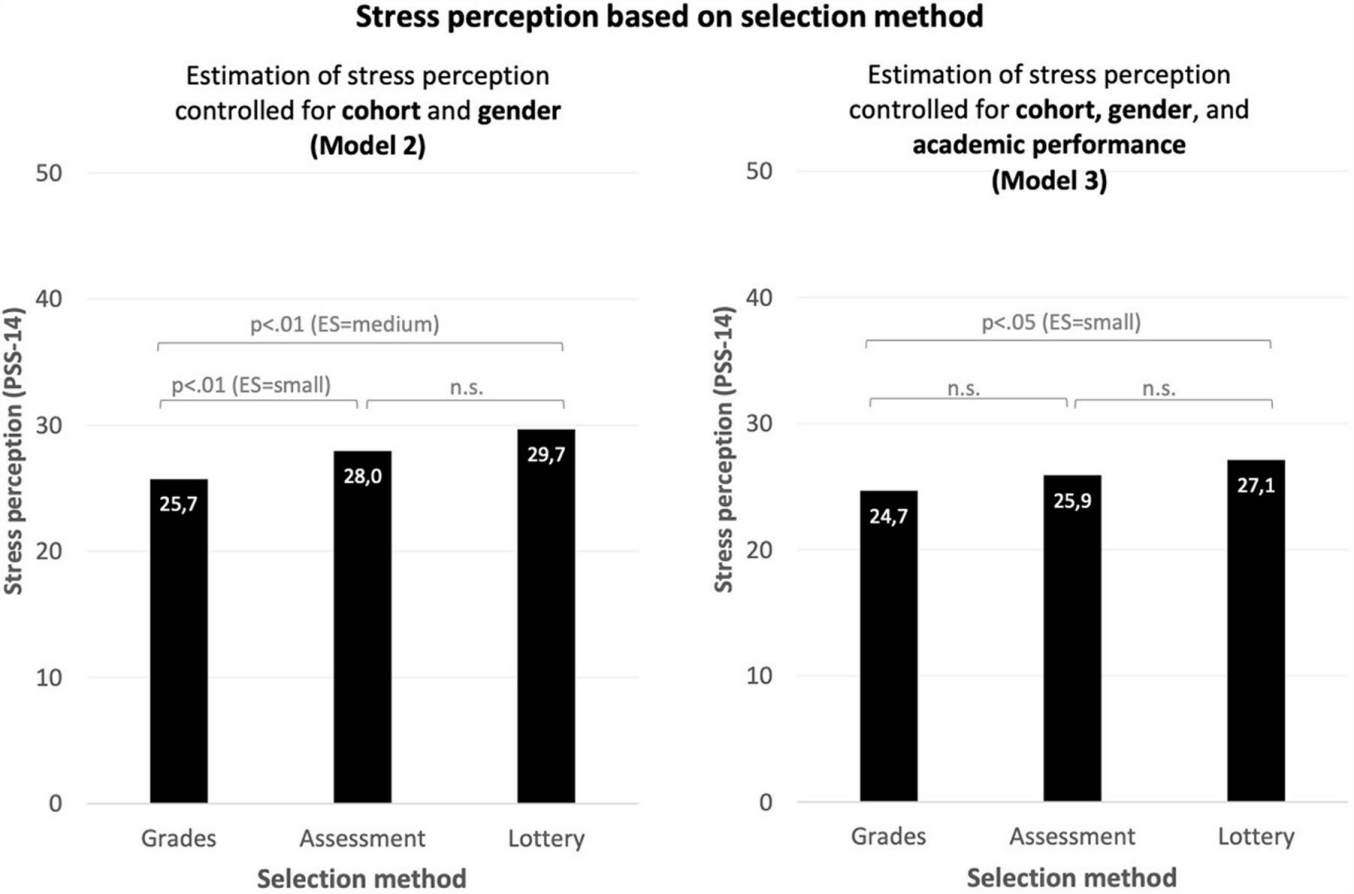
Estimated stress perception levels for each selection method Estimation stress perception levels, p-values and effect sizes (ES) are based on the results of the multilevel Model 2 and Model 3 as depicted in Table 2. Estimations are based on female students with (for Model 3) optimal academic performance. To interpret the figures for male students, the estimated stress perception level can be reduced by 3.63 (Model 2) and 3.66 (Model 3). For Model 3, to interpret the figure for non-optimal academic performance, estimated stress perception levels can be increased by 4.38

Generally, female students had a stress perception level that was 3.58 units higher compared to male students (B = 3.58 [2.23–4.94], *p* < .001, Table 2 – Model 1). Additionally, stress perception levels differed between cohorts (variance (SD) = 3.39 (1.84), Table 2 – Model 1). The cohort with the strictest performance standard, cohort 2014, showed the highest stress perception level. The model statistically significantly improved by adding selection method to the multilevel model (*p* < .01, Table 2 – Model 2). Students selected by assessment (B = 2.25 [0.84–3.66], ES = 0.3 *(small)*, *p* < .01) or weighted lottery (B = 3.95 [1.49–6.42], ES = 0.5 *(medium)*, *p* < .01) had higher stress perception levels compared to students selected by high grades (Table 2 – Model 2). Stress perception levels between students who were selected by weighted lottery or assessment did not statistically significantly differ. Estimations based on the multilevel model show that female students selected by high grades had stress perception levels of 25.7 (22.08 + 3.63), those selected by assessment 28.0 (22.08 + 3.63 + 2.25), and those selected by weighted lottery 29.7 (22.08 + 3.63 + 3.95; Table 2 – Model 2, Fig. 1 – Model 2).

### Post-hoc analysis: academic performance

In Model 3 (Table 2), Model 2 was extended by adding academic performance. Students with optimal academic performance had statistically significantly lower stress perception levels than students with non-optimal academic performance (B=-4.38 [-5.64 – -3.12, ES = 0.5 *(medium)*, *p* < .001,). As illustrated by Fig. 1, the difference between students selected by assessment and high grades was no longer statistically significant, and the difference between weighted lottery and grades decreased from 3.95 (ES = medium) to 2.45 (B = 2.45 [0.03–4.88], ES = 0.3 *(small)*, *p* < .05, Table 2 – Model 3).

**Table 2:** Multilevel models: Stress perception estimated by cohort, gender, selection and academic performance

		Stress perception in Year-1					
	Source of variation	Model 1		Model 2		Model 3 (post-hoc)	
		Coefficient	95% CI	Coefficient	95% CI	Coefficient	95% CI
**Fixed**	**(intercept)**	23.63	21.25–26.01	22.08	19.13–25.03	25.39	21.91–28.87
	**Gender**						
	Male	[reference]		[reference]		[reference]	
	Female	3.58^***^	2.23–4.94	3.63^***^	2.29–4.97	3.66^***^	2.37–4.96
	**Selection**						
	High grades			[reference]		[reference]	
	Weighted lottery			3.95^**^	1.49–6.42	2.45^*^	0.03–4.88
	Assessment			2.25^**^	0.84–3.66	1.22	-0.18–2.63
	**Academic performance**						
	Non-optimal					[reference]	
	Optimal					-4.38^***^	-5.64 – -3.12
**Random**	**Cohort (intercept)**						
	2013	-1.76		-2.50		-2.96	
	2014	1.78		1.68		2.11	
	2018	-0.02		0.82		0.84	
	**Variance (SD)**	3.39 (1.84)		5.19 (2.28)		7.26 (2.70)	
**Model**	**AIC**	4542.3		4533.3		4490.5	
	**Log L**	-2267.2		-2260.6		-2238.3	
	**p-value**	-		p < .01		p < .001	

^***^ p < .001, ^**^p < .01, ^*^p < .05

## Discussion

A variety of selection methods can be valuable given the quest of medical schools to create a well-performing and diverse student population. Although the prevalence of mental distress among medical students is high [1, 2], little is known about the association between different selection methods and the well-being of students attending medical school. The current study shows that the selection method is associated with the stress perception level of medical students in Year-1 of medical school. Our findings demonstrated that students selected by assessment or weighted lottery had higher stress perception levels in Year-1 than those selected by high pre-university grades. These higher stress levels were associated with non-optimal academic performance. Stress perception levels between students selected by weighted lottery or assessment did not statistically significantly differ.

The present study illustrates a gap in stress perception levels in Year-1 between students selected by high grades, who have lower stress levels, and students selected by assessment or weighted lottery. Controlling for academic performance closed the gap with students selected by assessment and narrowed the gap with students selected by weighted lottery. Thereby we confirm previous findings on the positive association between high grades-based selection and academic performance [18–20] as well as on the negative association between academic performance and stress [14, 15]. The present study adds the relevance of considering subgroups based on selection methods in relation to stress perception and the role of academic performance in this association.

The effect sizes of the present study show that the differences between differently selected students are small to medium. Although the PSS-14 is a widely used tool to compare groups and detect subgroup differences [35], the practical meaning of these effect sizes is hard to determine. Of note, PSS-values in the current study were comparable to other studies with students in European countries [39]. Furthermore, whether worse performance of students selected by assessment and lottery leads to higher stress perception levels or the other way around cannot be concluded from the present study. The finding that academic performance (partly) explains different stress levels among differently selected students requires additional research into the sequential order and underlying mechanisms of this association. Nevertheless, our results indicate that the link with the selection method may be a relevant starting point for further research.

A possible explanation for the finding that when controlling for academic performance, the gap was closed between the stress levels perceived by students selected by high grades and students selected by assessment, can be found in the Job Demands-Resources model (JDR model). The JDR model states that higher demands and lower resources lead to increased stress responses and decreased well-being [40, 41]. Non-optimal academic performance can be seen as a signal that the demands exceed the resources. It could be speculated that the resources of the students selected by high grades are better aligned with the demands of medical school. The competencies needed to achieve high pre-university grades may be similar to the competencies needed to perform well in Year-1 of medical school. Hence, these students are already used to what is being demanded. In contrast, the competencies of students selected by assessment, which is partly focused on extracurricular activities, may be of high value in becoming a medical doctor but are not fully rewarded in Year-1 of medical school and/or will have to be partly further developed in order to perform well. These students must adapt and/or develop resources to meet the demands. Furthermore, given that students are likely to continue these extracurricular activities while studying [42], it might even cause a conflict for them between life domains, resulting in enhanced stress perception [43] and lower academic performance [44, 45]. Thus, the difference in stress perception levels between students selected by high grades and assessment might be explained by a better alignment between the resources of the students and the demands of medical school for students selected by high grades compared to students selected by assessment.

For students selected by weighted lottery, stress perceptions remained statistically significantly higher compared to those of students selected by high grades after controlling for academic performance, although the magnitude of this effect decreased. In addition to the JDR model, an explanation for this finding can be sought in academic and social integration, as described by Tinto [46]. Academic integration refers to intellectual development and performance outcomes, whereas social integration refers to interactions with peers and faculty. Perhaps, students selected by high grades were academically better prepared given their high pre-university grades, which made it easier for them to integrate academically. However, the present study showed that even when students selected by weighted lottery perform optimally, they still have higher stress perception levels than students selected by high grades who perform optimally. It could be that social integration plays a role here besides academic integration. Social integration into the academic environment with its implicit social rules, values, and rituals, the so-called hidden curriculum [47, 48], may be easier for the probably more homogeneous group of students selected by high grades [17, 21]. It could be harder for students selected by lottery to, for example, feel connected with other students or find a mentor in the medical school they can identify with. Future research could elaborate on both academic and social integration as potential explanations to assess to which degree this plays a role in the elevated stress perception levels of students selected by lottery.

Several strengths and limitations of the present study should be considered. A strength is that multiple student cohorts were included, leading to a large sample size. The included cohorts were, however, several years back in time. A limitation is that different assessment and selection policies were active in the cohorts, but we controlled for these differences with multilevel analysis. The lottery group in the present study was relatively small compared to the high grades and assessment group but still large enough (n = 56) to draw meaningful conclusions. Finally, the present study was set up in an exploratory way since the association between the selection method and stress or any other variable related to the well-being of medical students has not been investigated previously. The explorative nature of the present study combined with the fact that it was executed in a single institution, warrants caution with generalization of findings to other institutions or settings. In addition, follow-up studies are necessary to confirm the associations found.

This exploratory study results in many new questions that can be addressed in future research, with a focus on unravelling the origin and underlying mechanisms of the different stress perception levels of differently selected students. For instance, it could be identified whether the different stress perception levels observed in the present study arose during medical school or whether these differences were pre-existent. In that respect, it would also be interesting to longitudinally determine the stress perception levels of differently selected students during their attendance at medical school. These longitudinal measurements could provide more insight into how stress levels of differently selected students develop during the pre-clinical and clinical phase of medical school.

Although the present study leaves many follow-up questions, it does offer some practical implications for medical schools to promote the well-being of all their students selected by various methods. In light of the theoretical explanations provided, alignment of their selection method with the medical school program and profession could play a role in this [17, 49], considering this positively impacts predictive value for performance in medical school [22]. The present study’s findings suggest that medical schools currently do not entirely succeed in this for students selected by assessment or weighted lottery. The literature describes two main discourses regarding improving student outcomes in higher education [50], which could also be applied to the well-being of students. The first - more dominant - discourse focuses on what institutions do to make students fit in the current academic environment (i.e., assimilation) [50]. An example would be to help students gain self-regulated learning skills that will help them to perform better, thereby increasing the students’ resources and positively contribute to their academic integration. For instance, the Study Smart program was shown to positively affect learning behaviour and academic performance [51]. The second discourse is focused on adjusting the academic environment for the increasingly diverse student population (i.e., accommodation) [50]. Schools could, for example, make changes to the learning environment by offering holistic assessments and stimulating that students are valued by the resources they do bring instead of focusing on resources they lack, so treat them as students “with” instead of students “without” [52, 53]. Thus, depending on the ideology and reasons for medical schools to use specific selection methods, they can organize additional support and/or adjust their curriculum and learning environment for students who enter medical school in other ways than with high grades.

## Conclusions

Selection methods intended to create a more diverse student population – assessment and lottery – are associated with higher stress perception levels in Year-1 of medical school. In addition, these higher stress perception levels are associated with non-optimal academic performance. However, even when controlling for academic performance, students selected by lottery still showed higher stress perception levels. To secure student well-being and, at the same time, not harm student diversity, more research is needed to determine why students selected by assessment and lottery have higher stress perception levels than those selected by high grades. In anticipation, the findings of the present study should encourage medical schools to critically consider the alignment between their selection criteria and the medical school program. In this way, some of the weight caused by a possible misalignment between selection methods and the medical school program can be transferred from the shoulders of medical students to the broader shoulders of the medical school itself.

## Electronic supplementary material

Below is the link to the electronic supplementary material.

Additional file 1: Overview of selection methods

Additional file 2: STROBE statement

## Data Availability

The database contains levels of perceived stress linked to students personal characteristics. We chose to not make these data publicly available due to the sensitivity of the data. Anonymized data is available upon request. Data requests may be sent to the institute of Medical Education Research Rotterdam (iMERR). Requests can be sent to scientific director Andrea Woltman (iMERR): a.woltman@erasmusmc.nl. iMERR can also be contacted via the website: www.imerr.nl, under the tab “Contact”.
