# Supplementary material for: The association between how medical students were selected and their perceived stress levels in Year-1 of medical school
Source: BMC Med Educ. 2023 Jun 16;23:443. doi: 10.1186/s12909-023-04411-0 (PMC10276376; doi:10.1186/s12909-023-04411-0)
Supplement: Supplementary file 1 — Additional file 1: Overview of selection methods [file 12909_2023_4411_MOESM1_ESM.docx]

**Additional file 1**

**Table. Overview of selection methods for cohort 2013, 2014 and 2018 at Erasmus MC Medical School including percentages of students admitted through a specific method.^*^**

|  | **High grades^†^** | **Assessment** | **Weighted lottery** |
| --- | --- | --- | --- |
| **Cohort 2013** | 12% | 66% | 22% |
| **Cohort 2014** | 28% | 64% | 8% |
| **Cohort 2018** | 69% | 31% | 0% |

^*^ Excluding students admitted through alternative routes (5-10% of each cohort). These alternative routes mainly consisted of students selected through participation in a scientific pre-university program and some exceptional cases due to personal circumstances. **^†^** Cohort 2013: selection by high grade if average grade of ≥8/10 based on *all* pre-university subjects. Cohort 2014: selection by high grade either if average grade of ≥8/10 based on *all* pre-university subjects, or average grade ≥7.5/10 on subjects Dutch, English, mathematics, physics, chemistry, and biology and sufficient motivation. Cohort 2018: selection by high grade, conditional on sufficient motivation, if average grade ≥7.5/10 on subjects Dutch, English, mathematics, physics, chemistry, and biology.
