# Supplementary material for: The association between how medical students were selected and their perceived stress levels in Year-1 of medical school
Source: BMC Med Educ. 2023 Jun 16;23:443. doi: 10.1186/s12909-023-04411-0 (PMC10276376; doi:10.1186/s12909-023-04411-0)
Supplement: Supplementary file 2 — Additional file 2: STROBE statement [file 12909_2023_4411_MOESM2_ESM.doc]

STROBE Statement—Checklist of items that should be included in reports of ***cohort studies***

|  | Item No | Recommendation | Our comment |
| --- | --- | --- | --- |
| **Title and abstract** | 1 | (*a*) Indicate the study’s design with a commonly used term in the title or the abstract | In the abstract, we indicate the study design: a retrospective multi-cohort study |
|  |  | (*b*) Provide in the abstract an informative and balanced summary of what was done and what was found | See Abstract |
| Introduction | | |  |
| Background/rationale | 2 | Explain the scientific background and rationale for the investigation being reported | See the Introduction |
| Objectives | 3 | State specific objectives, including any prespecified hypotheses | In the Introduction, we mention the research question: Do differences exist in stress perception levels at the end of Year-1 between medical students selected on the basis of high grades, assessment, or weighted lottery?  Since this is an exploratory study, as indicated in the Introduction, no hypotheses are specified. |
| Methods | | |  |
| Study design | 4 | Present key elements of study design early in the paper | In the Introduction, we state that it concerns a retrospective multi-cohort study. In addition, we aimed to present key elements of study design in the methods section by describing the context, participants and procedure, measurements and analysis steps. |
| Setting | 5 | Describe the setting, locations, and relevant dates, including periods of recruitment, exposure, follow-up, and data collection | Under the subsection “context”, we describe the setting and location of the study. Under the subsection “participants and procedure” we describe the dates for data collection. |
| Participants | 6 | (*a*) Give the eligibility criteria, and the sources and methods of selection of participants. Describe methods of follow-up | Under the subsection “participants and procedure”, the eligibility criteria are described. |
|  |  | (*b*) For matched studies, give matching criteria and number of exposed and unexposed | Not applicable |
| Variables | 7 | Clearly define all outcomes, exposures, predictors, potential confounders, and effect modifiers. Give diagnostic criteria, if applicable | Included variables in this study are:  - Outcome variable: stress perception level  - Predictors: selection method, cohort/assessment policy, gender, and academic performance.  Selection method as well as assessment policy are described under “context”.  Student cohort and gender are described under “participants and procedure”.  Under the subsection “Measurements” we define the variables stress perception level and academic performance. |
| Data sources/ measurement | 8* | For each variable of interest, give sources of data and details of methods of assessment (measurement). Describe comparability of assessment methods if there is more than one group | See point 7 above. |
| Bias | 9 | Describe any efforts to address potential sources of bias | As described in the “Analyses” subsection:  The sample of students who completed the PSS-14 questionnaire was compared to the complete cohort with chi-square tests to assess the representability of the sample. |
| Study size | 10 | Explain how the study size was arrived at | As described in the “Participants and procedures” subsection: The number of students that completed the questionnaire and provided informed consent determined the sample size of the study. |
| Quantitative variables | 11 | Explain how quantitative variables were handled in the analyses. If applicable, describe which groupings were chosen and why | Perceived stress was the only quantitative, numeric variable and was handled as such as outcome. No groupings were chosen for this variable. |
| Statistical methods | 12 | (*a*) Describe all statistical methods, including those used to control for confounding | a+b) See “Analyses” subsection  c) Only complete questionnaires were included, so no missing data  d+e) Not applicable |
|  |  | (*b*) Describe any methods used to examine subgroups and interactions |  |
|  |  | (*c*) Explain how missing data were addressed |  |
|  |  | (*d*) If applicable, explain how loss to follow-up was addressed |  |
|  |  | (*e*) Describe any sensitivity analyses |  |
| Results | | |  |
| Participants | 13* | (a) Report numbers of individuals at each stage of study—eg numbers potentially eligible, examined for eligibility, confirmed eligible, included in the study, completing follow-up, and analysed | See Table 1 |
|  |  | (b) Give reasons for non-participation at each stage | Inclusion of participants is described in the subsection “Participants and procedure” (Methods section). There were no different stages in the data collection. |
|  |  | (c) Consider use of a flow diagram | Not applicable |
| Descriptive data | 14* | (a) Give characteristics of study participants (eg demographic, clinical, social) and information on exposures and potential confounders | See Table 1 |
|  |  | (b) Indicate number of participants with missing data for each variable of interest | Not applicable |
|  |  | (c) Summarise follow-up time (eg, average and total amount) | Not applicable |
| Outcome data | 15* | Report numbers of outcome events or summary measures over time | Not applicable |
| Main results | 16 | (*a*) Give unadjusted estimates and, if applicable, confounder-adjusted estimates and their precision (eg, 95% confidence interval). Make clear which confounders were adjusted for and why they were included | See Table 2 |
|  |  | (*b*) Report category boundaries when continuous variables were categorized | Not applicable |
|  |  | (*c*) If relevant, consider translating estimates of relative risk into absolute risk for a meaningful time period | Not applicable |
| Other analyses | 17 | Report other analyses done—eg analyses of subgroups and interactions, and sensitivity analyses | Not applicable |
| Discussion | | |  |
| Key results | 18 | Summarise key results with reference to study objectives | See first part of the Discussion (up until strengths and limitations) |
| Limitations | 19 | Discuss limitations of the study, taking into account sources of potential bias or imprecision Discuss both direction and magnitude of any potential bias | See paragraph on strengths and limitations. |
| Interpretation | 20 | Give a cautious overall interpretation of results considering objectives, limitations, multiplicity of analyses, results from similar studies, and other relevant evidence | See first part of the Discussion (up until strengths and limitations) |
| Generalisability | 21 | Discuss the generalisability (external validity) of the study results | See paragraph on strengths and limitations in the Discussion. |
| Other information | | |  |
| Funding | 22 | Give the source of funding and the role of the funders for the present study and, if applicable, for the original study on which the present article is based | Not applicable |

*Give information separately for exposed and unexposed groups.

**Note:** An Explanation and Elaboration article discusses each checklist item and gives methodological background and published examples of transparent reporting. The STROBE checklist is best used in conjunction with this article (freely available on the Web sites of PLoS Medicine at http://www.plosmedicine.org/, Annals of Internal Medicine at http://www.annals.org/, and Epidemiology at http://www.epidem.com/). Information on the STROBE Initiative is available at http://www.strobe-statement.org.
